# In-hospital moderate intensity interval training following surgical resection of foregut malignancy – a prospective single arm feasibility study

**DOI:** 10.1007/s00520-026-10453-z

**Published:** 2026-02-24

**Authors:** Michael W. Hii, Rosalind Walmsley, Qianyu Chen, Lynn Chong

**Affiliations:** 1 Department of Surgery, St Vincent’s Hospital Melbourne, The University of Melbourne, Melbourne, Australia; 2 Department Upper GI and HPB Surgery, St Vincent’s Hospital Melbourne, 41 Victoria Pde, Fitzroy, 3065 Australia

**Keywords:** Foregut, Interval training, Gastrectomy, Hepatectomy, Esophagectomy

## Abstract

**Background:**

Surgical resections of foregut malignancy are generally procedures with substantial morbidity and mortality. The use of exercise therapy has resulted in improvements in outcomes in many parts of the cancer treatment pathway and thus has become a standard addition to oncology and surgical oncology paradigms including during chemotherapy and as prehabilitation and rehabilitation for surgery. Guided in-hospital interval training as post-surgical therapy has not been evaluated as a treatment tool. If safe and acceptable to patients, this has the potential to improve surgical outcomes.

**Methods:**

Twenty-one subjects were enrolled in a prospective single-arm trial of guided in-hospital, immediately postoperative moderate- (+) intensity interval training while recovering from a resection of foregut malignancy. A specific exercise program was designed for the postoperative setting and administered, aimed at achieving maximal intensity training during surgical recovery in the hospital environment. We hypothesized that this intervention would be safe, acceptable to patients, and would not negatively influence health-related quality of life. This was assessed by compliance with exercise sessions, the incidence of training-related complications, and the EORTC QLQ-30 and Hospital Anxiety and Depression scores.

**Results:**

Twenty-one patients were enrolled in this study (7 esophagectomies, 10 laparotomies, 4 laparoscopies). Participation in the program was high with 90.5% of subjects completing greater than 25% of all possible sessions and 71.4% of patients completing greater than 50% of possible sessions. There was no intervention-related adverse event. There was no reduction in emotional quality of life measures on discharge.

**Conclusions:**

Administering a postoperative moderate- (+) intensity interval training program following recovery from major foregut surgery is acceptable to patients with high rates of participation. It is safe and does not result in a reduction in emotional health related quality of life.

**Supplementary information:**

The online version contains supplementary material available at 10.1007/s00520-026-10453-z.

## Novelty statement

1. Key findings
  Guided postoperative, in-hospital physical therapy with an interval training program is safe and acceptable to patients and does not result in any reduction in mood nor emotional quality of life.
2. What is known and what is new?
  Immediate postoperative in-hospital interval training programs have not been evaluated as a clinical tool.
  This manuscript confirms the feasibility and safety of implementing an interval training program following major foregut surgery.
3. What is the implication, and what should change now?
  The feasibility data from this study will form the basis of a randomized trial to assess the clinical benefits of administering interval training after surgery in this patient group (Trial ID ACTRN12620000315910.

## Introduction

Regular exercise improves physical capacity and health outcomes independently of age, sex, or the presence of chronic disease [1]. Exercise has become an important adjunct to standard treatment in oncology and surgical oncology [2, 3]. Significant and clinically meaningful positive effects on health-related quality of life and cardiorespiratory fitness have been demonstrated in randomized trials and meta-analyses which have assessed the role of exercise during chemotherapy, prehabilitation, and rehabilitation after surgery [2, 4–6]. This has led to exercise being recommended as part of standard care by the Clinical Oncological Society of Australia and other national and international health organizations [7, 8].

Cancer treatment often involves major surgery which is associated with a risk of morbidity and mortality and can also have a substantial negative functional impact on patients. For instance, after oesophagectomy, return to baseline quality of life typically takes a median of 12 months [9]. Given enough time, optimization of physical fitness prior to surgery can improve perioperative outcomes [4, 10–12]. In patients undergoing surgical resection of malignant disease, preoperative exercise therapy can improve cardiorespiratory fitness and also postoperative outcomes including surgical morbidity, hospital length of stay, and quality of life [4, 10, 13–15]. However, in many situations, the duration between diagnosis of cancer and the commencement of treatment is necessarily brief, hence the interest in identifying other opportunities to improve physical function.

Following surgical resection of foregut malignancies, there is often a relatively extended hospital length of stay [16]. This time, in-hospital may be an occasion to administer exercise therapy with potential for benefits in both short-term and long-term patient recovery. In the postoperative setting, enhanced recovery programs are now standard elements of care; however, there is a lack of evidence to guide the specific timing and format of physical activity recommended to patients to optimize recovery [17]. Data from bariatric, orthopedic, cardiac, and thoracic surgery literature suggests that very early implementation of postoperative rehabilitation may be beneficial [18–21].

High-intensity interval training (HIIT) is a popular form of concentrated exercise activity. HIIT is not universally defined but is characterized by repeated short bursts of intense activity, performed with a “near maximal” or “all-out” effort [1]. HIIT elicits greater reported enjoyment from exercise while inducing physical adaptations similar to or greater than moderate-intensity continuous training, despite a lower total exercise volume [22, 23]. In both clinical and healthy populations, HIIT has been shown to induce physiological adaptations including improving maximal oxygen uptake, aerobic endurance, anaerobic capacity and power, and functional capacity [22–24].

Given the constraints of the hospital ward, HIIT is a time- and space-efficient form of exercise, is particularly suitable to this setting; however, as defined, HIIT may not always be physically possible in a postoperative context due to post-surgical pain, nausea, fatigue or other symptoms. It is not known if it is safe to administer this kind of exercise in this setting.

We have therefore designed a feasibility study to assess an in-hospital, postoperative interval training program for patients undergoing resection of foregut malignancy. The primary objective was to demonstrate that in-hospital rehabilitation with interval training can be implemented, without adverse events and with good compliance to supplement the immediate postoperative recovery. We hypothesized that this intervention would be safe, acceptable to patients and would not negatively influence health-related quality of life. If this hypothesis was confirmed, this data would form the basis of a future randomized trial. We present this article in accordance with the TREND reporting checklist [25].

### Methods

A prospective, non-randomized, single-arm trial was performed to assess the feasibility, safety, and patient acceptability of an in-hospital interval training program after surgery for resection of foregut malignancy.

The study protocol was approved by the Human Research Ethics Committee at St Vincent’s Hospital, Melbourne prior to initiation (HREC. 119/19). Each participant was provided a thorough description of the purpose, protocols, and assessments in the trial prior to providing written informed consent. All procedures were conducted in accordance with the 1964 Helsinki Declaration.

The exercise prescription was designed by a registered exercise physiologist and administered by trained staff under the supervision of the participant’s nurse. The exercise program was designed to enable patients to reach the highest intensity they were able. Interval training was defined as intermittent exercise bouts performed at moderate or above intensity with intervening periods of rest [26]. The physiological target for HIIT was defined as a heart rate between 77 and 95% of theoretical maximum (220—age in years) or a perceived rating of physical exertion (RPE) of seven to nine (out of 10) [27]. Moderate intensity was defined as 64–76% theoretical maximum heart rate or RPE of five to seven (out of 10) [27]. This study was designed to assess the real-world application of interval training therapy and while the aim was to achieve physiological targets of HIIT, it was recognized that this may not be possible for each subject in each session in the postoperative environment. It was expected that the maximum intensity reached in each session would vary according to an individual’s recovery trajectory. As such, if a patient started an exercise session, this was included as trial participation regardless of maximum intensity achieved.

The exercise program involved the use of dumbbells (up to 5 kg), ankle weights (1.5 kg), a step and a pedal exerciser. Lying, sitting and standing variations of each exercise were available, and the program was applied and scaled according to each participant’s postoperative capacity such that it did not exacerbate pain or any other symptom. Exercise was ceased immediately if the participant reported experiencing unpleasant symptoms during the intervention. Each day, one aerobic and one resistance session were offered with at least a 2-h interval between. Aerobic exercise included five to 20 min of training performed in intervals using either in-bed or seated cycling, step ups, or walking. Intervals varied from 10 s to 1 min with either active or passive recovery. Resistance exercise involved two to five exercises targeting major upper and lower body muscle groups. Intensity was manipulated from six to 15 repetitions and one to three sets per exercise. There was a minimum of 1 min-rest between exercise sets. Example exercise options are shown in Table 1, and the full program is listed in appendix 1.

**Table 1:**
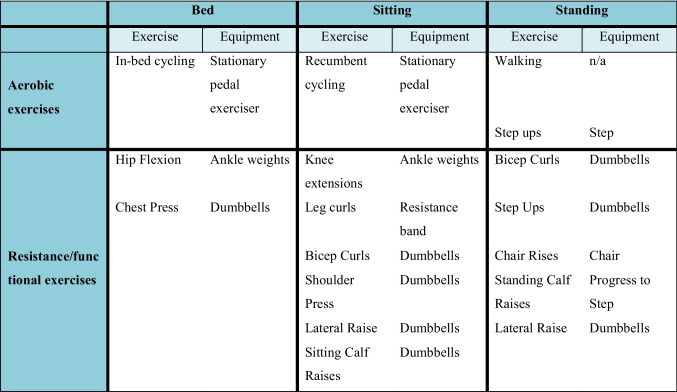
Example exercise options

Participants were guided to focus on technique including movement, speed, posture and coordination with sufficient rest in-between to allow for recovery. Exercise intensity was measured using the participants’ heart rate during exercises and a subjective assessment using the modified Borg scale to rate self-perceived levels of exertion [28]. Patients were encouraged to exercise within their own perceived limits with high intensity being the goal. The exercise prescription was progressive and modified according to individual response and recovery status. Participants were clearly explained not to push exercise capacity at the expense of increasing pain or other postoperative symptoms. If high intensity was beyond a subject’s capacity, lower levels of exercise were accepted.

Exercise was offered to participants on postoperative day 1 and then each day until discharge or day 14 was reached (weekends and public holidays were omitted for staff availability). Participants that experienced a serious surgical complication (Clavien-Dindo 3 +) were withdrawn from the exercise program, but data included on an intention-to-treat basis [29].

There is no previous data to inform a power calculation and so our study aimed to enrol 21 participants at a single tertiary university hospital. Consecutive patients scheduled for resection of foregut malignancy were offered enrolment. Patients were included if they were 18 years or older and able to understand and follow exercise instructions in English. Foregut malignancy was defined as esophageal, gastric, duodenal, pancreatic, biliary or hepatic tumor or carcinoma.

Patients were excluded in the event of emergency procedures, multiple malignancies, or uncontrolled medical co-morbidities that would preclude exercise therapy, such as severe ischaemic heart disease or chronic obstructive pulmonary disease.

We aimed to include a broad range of surgical types. Surgeries were classified into three groups based on the relative trauma of access:

- Abdominal surgery via laparotomy
- Abdominal surgery via laparoscopy
- Esophagectomy

Anesthetic, surgical, and standard postoperative care protocols were based on the institution’s existing policies without variation other than the addition of exercise therapy.

Outcome measures with time points are shown in Table 2.

**Table 2:** Outcome measures

	Study period				
Time points	Enrolment	Baseline	Postoperative		
Outcome measure			Day of surgery	In-hospital stay	Discharge
Eastern Cooperative Oncology Group (ECOG) status	X				
Medical history; demographics	X				
Anthropometry	X				
EORTC*—QLQ C30		X			X
Hospital Anxiety and Depression Score (HADs)		X			X
6-min walk test		X			X
Grip strength		X			X
Borg rating of perceived exertion				X	
American Society of Anaesthesiology Score			X		
Heart rate				X	
Length of hospital stay					X
Postoperative complications				X	X
Adverse events related to intervention			X	X	X

^*^EORTC, European Organisation for Research and Treatment of Cancer—scores calculated based on the EORTC scoring manual

For the purposes of this study, patient acceptability was defined as a patient participating in > 25% of possible exercise sessions. Safety was defined as the absence of exercise-induced adverse events. Attrition was defined as a participant withdrawing consent or asking to be removed from the trial.

Study data was collected and managed using REDCap (Research Electronic Data Capture) electronic data capture tools hosted at the University of Melbourne [30, 31].

As a feasibility study, we report descriptive data on baseline demographics, operation types, ECOG status, and quality of life (QoL) scores. This data is summarized by using means and standard deviation or median and interquartile ranges (for continuous variables) or frequencies and percentages (for categorical variables), as appropriate. We used one-way analysis of variance (ANOVA), Kruskal-Wallis and chi-squared test to compare patients’ participation rate in each type of exercise session, earliest day of participation and rate of exercise-related adverse events across different surgery types. Matched student’s *T*-test was used to compare each patient’s functional fitness and reported QoL scores on discharge against their own at baseline. Statistical significance was accepted at a *p*-value less than 0.05.

A discharge interview was conducted with participants, with comments collated and presented in diagrammatic form.

## Results

Twenty-one patients were recruited into the study between August and December of 2019, with all participants commencing exercise therapy. Participant baseline characteristics, oncology diagnosis and surgical details are shown in Table 3.

**Table 3:** Baseline characteristics

	All patients (*n* = 21)
Demographics	
Sex *n* (%)	
Female	9 (42.9)
Male	12 (57.1)
Age mean (SD)	68.4 (11.1)
Anthropometrics	
Height (cm) mean (SD)	170 (11.9)
Weight (kg) mean (SD)	76.2 (15.3)
ECOG score median (IQR)	0 (0)
ASA* score median (IQR)	3 (1)
Diagnosis *n* (%)	
Esophageal malignancy	6 (28.6)
Squamous cell carcinoma	2 (28.6)
Adenocarcinoma	4 (57.1)
Esophagogastric junction malignancy	1 (4.8)
Adenocarcinoma	1 (4.8)
Gastric malignancy	3 (14.3)
Gastrointestinal stromal tumor	2 (9.5)
Adenocarcinoma	1 (4.8)
Liver malignancy	7 (33.3)
Primary hepatocellular carcinoma	1 (4.8)
Colorectal cancer metastases	6 (28.5)
Cholangiogarcinoma	2 (9.5)
Pancreatic adenocarcinoma	2 (9.5)
Procedure type *n* (%)	
Esophagectomy	7 (33.3)
Ivor Lewis (laparotomy, thoracotomy)	2 (9.5)
Three-stage (thoracoscopy, laparotomy)	5 (23.8)
Laparotomy	10 (47.6)
Pancreatic-duodenectomy (Whipple)	4 (19.0)
Liver resection	4 (19.0)
Gastrectomy (radical subtotal)	1 (4.8)
Gastrectomy (partial)	1 (4.8)
Laparoscopic	4 (19.0)
Liver resection	3 (14.3)
Partial gastrectomy for GIST	1 (4.8)

^*^ASA, American Society of Anaesthesiologists

Surgical outcome data is shown in appendix 2. Two participants experienced a severe complication (Clavien Dindo Grade 3+)—both were anastomotic leaks in the esophagectomy arm and were unrelated to the exercise intervention. These two participants ceased participation in the exercise program after this complication. There were no complications above Clavien Dindo grade 3.

Feasibility data is shown in Table 4. There was a high level of participation with exercise beginning on a median of postoperative day 1, 60% of total possible sessions were completed by participants, and 91% of participants achieved the pre-defined “patient acceptability” target of 25% of exercise sessions. A mean of 10 sessions was completed per participant. No participant asked to be removed from the study or withdrew consent and no adverse event relating to participation in this study was recorded. Details of each patient’s average heart rate and rating of perceived exertion can be found in appendix 3.

**Table 4:** Feasibility of interval training program by surgery type

	Oesophagectomy (*n* = 7)	Laparotomy (*n* = 10)	Laparoscopic (*n* = 4)	All patients (*n* = 21)	*p*-value
Total sessions per patient *Mean (SD)*	10.3 (3.3)	11.4 (4.0)	5.8 (3.1)	10.0 (3.9)	**0.037**
Postoperative day * of commencement *Median (IQR)*	1 (1)	1 (2)	3 (4)	1 (1.5)	0.058
% of possible sessions completed *Mean (SD)*	71.0 (21.7)	64.4 (21.1)	29.2 (25.0)	59.9 (26.0)	**0.019**
% resistance sessions *Mean (SD)*	62.9 (25.6)	56.1 (22.5)	22.9 (20.8)	52.1 (26.6)	**0.036**
% aerobic sessions *Mean (SD)*	33.0 (8.5)	44.1 (21.3)	6.3 (12.5)	33.2 (21.3)	**0.005**
Participation in any session					
Patients participating in any session *n (%)*	7 (100)	10 (100)	3 (75)	20 (95.2)	0.107
Patients participating in any session—resistance *n (%)*	7 (100)	10 (100)	3 (75)	20 (95.2)	0.107
Patients participating in any session—aerobic *n (%)*	7 (100)	10 (100)	1 (25)	18 (85.7)	**0.001**
Participation in > 25% possible sessions					
Patients participating in ≥ 25% sessions—overall *n (%)*	7 (100)	10 (100)	2 (50)	19 (90.5)	**0.009**
Patients participating in ≥ 25% sessions—resistance *n (%)*	7 (100)	9 (90)	2 (50)	18 (85.7)	0.064
Patients participating in ≥ 25% sessions—aerobic *n (%)*	6 (85.7)	7 (70)	1 (25)	14 (66.7)	0.115
Participation in > 50% possible sessions					
Patients participating in ≥ 50% sessions—overall *n (%)*	6 (85.7)	7 (70)	2 (50)	15 (71.4)	0.447
Patients participating in ≥ 50% sessions—resistance *n (%)*	5 (71.4)	6 (60)	1 (25)	12 (57.1)	0.315
Patients participating in ≥ 50% sessions – aerobic *n (%)*	0 (0)	5 (50)	0 (0)	5 (23.8)	**0.027**
Participation in all sessions					
Patients participating in all sessions *n (%)*	1 (14.3)	1 (10)	0 (0)	2 (9.5)	0.738
Patients withdrawing consent *n (%)*	0 (0)	0 (0)	0 (0)	0 (0)	NA
Exercise-related adverse event *n (%)*	0 (0)	0 (0)	0 (0)	0 (0)	NA

Bold font, *p* < 0.05

^*^If surgery was performed on Friday, postoperative day 1 is defined as Monda

Functional and quality of life measures are shown at baseline and following the exercise intervention (i.e., on discharge) in Table 5 and 6, respectively. A significant reduction in right hand grip strength and 6-min walk test (6MWT) distance on discharge was recorded. This was consistent with the impact of major surgery [32]. Similarly, quality of life scores were worse in numerous physical domains also in keeping with the postoperative effect [33]. There was no change in mood related quality of life scores.

**Table 5:** Functional fitness at preoperative baseline and on discharge from hospital

	Baseline (*n* = 12)	On discharge (*n* = 9)	Change	*p*-value
Max. grip strength—left hand kg mean (SD)	29.5 (11.2)	29.8 (11.2)	−2.51 (3.07)	0.142
Max. grip strength—right hand kg mean (SD)	30.5 (12.0)	29.6 (10.8)	−3.83 (2.46)	**0.025**
6MWT distance completed (meters) mean (SD)	403.8 (153.8)	328.6 (74.0)	−110.7 (112.0)	**0.040**

Bold font, *p* < 0.05. *6MWT*, 6-min walk test

**Table 6:** HAD scale and EORTC score on admission and on discharge

	Admission (*n* = 15)	On discharge (*n* = 8)	Inter-patient change	*p*-value
**HAD score**				
Anxiety mean (SD)	4.7 (3.6)	4.6 (4.8)	0.3 (2.8)	0.733
Depression mean (SD)	2.6 (3.3)	2.6 (2.4)	−1 (2.5)	0.2645
**EORTC score**				
Global status mean (SD)	71.1 (26.5)	43.8 (26.6)	**−41.7 (22.3)**	**0.001**
Physical function mean (SD)	90.7 (12.3)	78.3 (15.0)	**−19.2 (16.9)**	**0.014**
Role function mean (SD)	74.4 (38.2)	45.8 (42.5)	−37.5 (46.1)	0.055
Emotional function mean (SD)	85.6 (23.7)	75 (37.5)	−11.9 (19.2)	0.151
Cognitive function mean (SD)	90.0 (18.7)	85.4 (24.3)	−8.3 (29.5)	0.451
Social function mean (SD)	94.4 (20.6)	77.1 (37.7)	−20.8 (38.6)	0.170
Fatigue mean (SD)	22.2 (20.2)	38.9 (21.4)	**26.5 (24.5)**	**0.019**
Nausea vomiting mean (SD)	6.7 (12.3)	10.4 (17.7)	2.3 (12.4)	0.197
Pain mean (SD)	12.2 (16.0)	31.2 (35.0)	28.6 (36.9)	0.087
Dyspnea mean (SD)	11.1 (16.3)	20.8 (35.3)	12.5 (35.4)	0.351
Insomnia mean (SD)	20.0 (24.6)	66.7 (35.6)	**37.5 (41.5)**	**0.038**
Appetite mean (SD)	11.1 (20.6)	41.7 (34.5)	**37.5 (27.8)**	**0.007**
Constipation mean (SD)	6.7 (13.8)	45.8 (39.6)	**45.8 (39.6)**	**0.014**
Diarrhea mean (SD)	2.2 (15.3)	12.5 (24.8)	14.3 (26.2)	0.200
Financial difficulties mean (SD)	4.4 (17.2)	0 (17.8)	0 (0)	NA

Bold font, *p* < 0.05

*HADS*, hospital anxiety and depression scale; *EORTC*, European Organisation for Research and Treatment of Cancer. Where higher linear score indicates higher (better) function and higher degree (worse) of symptoms

## Discussion

This feasibility study is the first to implement an immediate postoperative (in hospital) interval training program as part of physical rehabilitation. In this cohort, interval training was prescribed following resection of foregut malignancy. Our results show that interval training in this cohort is a safe therapy that is acceptable to patients and is not associated with any measurable negative influence on emotional health–related quality of life.

Recovery after surgery is often limited by postoperative pain and the surgical stress response and may be worsened by a reduction in mobilization and nutrition. There has been a widespread adoption of multimodal enhanced recovery after surgery (ERAS) programs with many specialties realizing an improvement in postoperative outcomes [34–37]. It is not known, however, which factor or which combination of factors in the ERAS programs delivers the key benefits. Although ERAS programs typically incorporate some form of exercise prescription, there is a lack of evidence to guide a specific understanding of the ideal type of postoperative mobilization and physical rehabilitation – that is when, how, and how much to implement as postoperative physical therapy [34].

HIIT is a relatively simple, time- and space-efficient therapy which has proven benefits in specific clinical groups and may improve recovery after surgery [3, 38]. There are few previous exercise intervention studies which have applied interval training in the immediate postoperative situation. We wanted to study the role of HIIT as an exercise intervention in this context; however, it was clear that HIIT may not be achieved during some of the intervention sessions in postoperative recovery phase. In this study, subjects were given instructions to exercise up to a HIIT level but also to allow lesser efforts in the interests of surgical safety. As such, this study evaluates moderate (+) intensity interval training in the hospital setting. This is a step as an early part of the assessment of the role of HIIT in surgical patients, and we postulate that this reflects real-world application of exercise intervention in a clinical environment.

In this study, we have chosen surgery for foregut malignancy as the interest group. This included patients undergoing laparotomy and esophagectomy where the hospital length of stay is typically longer compared to other gastrointestinal procedures and other minimally invasive procedures [39]. It is also thought that the surgical trauma and consequently physiological stress response are higher (and morbidity rate) in this surgical group in comparison to other gastrointestinal surgical procedures [39]. This allows a greater in-hospital stay for potential exercise interventions and greater scope for improvement in outcomes if this therapy is shown to be beneficial.

**Fig. 1:**
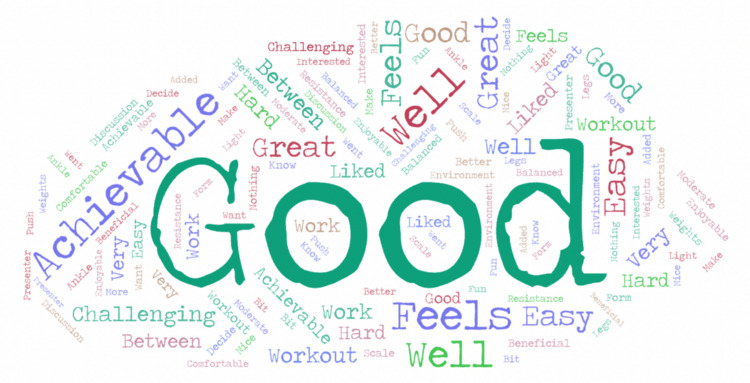
Word-cloud of patients’ comments

As with other novel interventions, inter-participant uptake varied but there was an overall high level of engagement. Patient interaction was favorable, and discharge interviews suggested a positive experience. HAD score and EORTC scores were recorded. There were expected postoperative changes in physical function quality of life scores, but importantly, there was no apparent reduction in emotional and mood health-related quality of life after surgery or exercise intervention, although no control group was available for comparison. We interpreted this to suggest that physical exercise did not negatively influence quality of life in these domains. This provides a baseline for a future comparison of health-related quality of life after exercise intervention with a control group in a future study. A discharge interview was conducted with patients regarding their exercise experience. A word cloud of patient comments is shown in Fig. 1.

This study was not powered to assess between surgery differences in uptake of exercise therapy, nor the impact of the exercise intervention on outcomes. A broad range of surgical procedures was included to support the hypothesis that this exercise intervention can be applied widely after different surgery types.

There are potential risks associated with interval training after surgery, particularly if this is aimed at working towards HIIT. Exercise injuries and discomfort (e.g., fall, muscle or joint strain) when performing exercise could occur. There is also a theoretical risk of adverse events, such as tearing of sutures or injury to a patient’s surgical wound. Using an experienced exercise physiologist to design this program and a commonsense approach to exercise selection, we assessed these risks as limited; however, to mitigate this small risk, each exercise session was administered by trained staff and monitored by medical and nursing members of the treating team. No adverse events relating to performing the interval training program were seen in this study.

With specific reference to surgery, previous research on the role of exercise therapy has focused on prehabilitation and post-discharge exercise programs [1, 4, 6, 10, 11, 13–15, 19, 21, 40–44]. Few studies have assessed exercise interventions in the immediate postoperative in-patient setting [17, 20, 45–48]. Further, aerobic interventions have generally been studied separately from resistance interventions. Previous nonclinical exercise programs have suggested that there are synergistic benefits to combining aerobic and resistance training [49, 50] 

Our results are in broadly in line with these previous studies which have looked at other kinds of early exercise in a post-surgical setting noting that previous data has not been conclusive in showing a meaningful benefit. In a non-randomized study, Bhatt et al. demonstrated the efficacy of a pedal exercise regime immediately after major abdominal surgery in reducing respiratory complication rates and length of hospital stay [51]. However, a randomized study from Fiore et al. which randomized patients undergoing colorectal cancer resection to assisted mobilization from postoperative day 0 to day 3 saw no improvement in any of the outcomes measured. This was despite a markedly increased step count in the intervention group [45].

Resistance training alone has previously been shown to be safe following surgery. Schram et al. published their experience implementing a resistance program after colorectal cancer resection (29/30 procedures laparoscopic) reporting similar findings of high patient engagement and no exercise-related adverse events [17]. A similar study performed in patients undergoing laparoscopic gastrectomy for early gastric cancer further supports the safety and feasibility of in-hospital postoperative resistance exercise [47].

Combining aerobic and resistance exercises immediately post-surgery has also been shown to be safe and beneficial. A low-intensity mixed exercise program using a randomized trial design by Ahn et al. demonstrated significant benefits in terms of return of flatus and length of stay after colectomy for colon cancer [48].

Thus, limited previous evidence supports in-hospital exercise rehabilitation as safe and feasible with potential benefit. In contrast to the training programs used in these studies, the current protocol involves a combination of aerobic and resistance training delivered in an interval format with the capacity to engage in high-intensity training where possible. HIIT is thought to create greater physical benefit per unit of exercise intervention over low to medium-intensity training [22, 24].

It is an advantage of this study’s design to incorporate multiple surgery types and approaches, including laparotomy and thoracotomy. This implies that postoperative interval training programs will be broadly acceptable in most postoperative settings. In addition, as a single center trial, we can be relatively certain that postoperative care protocols are standardized and consistent between participants. As such, we are confident in the safety of this program’s implementation. However, this study is not without limitations. Firstly, exercise therapy was not available on weekends and, as such, some shorter stay patients were only able to access one session. Secondly, despite efforts to communicate and educate clinical staff regarding the details of this study, some were disconcerted by the concept making implementation less straightforward for some patients. Finally, according to the definition, high intensity was not achieved in many of the exercise sessions. This may be seen as a limitation, but we feel this is a more real-world approach to exercise therapy, noting that even in non-clinical populations, HIIT is not always achieved during an exercise session despite intentions [52].

This study has demonstrated that a moderate (+) intensity interval training program, designed specifically for postoperative patients, can be implemented in a clinical setting safely and with high rates of patient engagement. It is not clear if this form of intensive rehabilitation can improve surgical outcomes. Implementing this therapy does encounter challenges, and careful attention is required to establish patient expectations and educate staff members involved in patient care. The feasibility data from this study will form the basis of a randomized trial to assess the clinical benefits of administering interval after surgery in this patient group (Trial ID ACTRN12620000315910).

## Conclusions

The use of moderate (+) intensity interval training after foregut cancer resection is safe, feasible, and has a high rate of patient acceptance. There is no apparent negative influence of this kind of training on emotional quality of life, anxiety, or depression on discharge from hospital.

## Supplementary information

Below is the link to the electronic supplementary material.

ESM 1 (DOCX 15.4 KB)

ESM 2 (DOCX 40.1 KB)

ESM 3 (PDF 216 KB)

ESM 4 (PDF 168 KB)

ESM 5 (PDF 169 KB)

## Data Availability

The study methods and materials will be made available on request to the corresponding author.
