# Supplementary material for: In-hospital moderate intensity interval training following surgical resection of foregut malignancy – a prospective single arm feasibility study
Source: Support Care Cancer. 2026 Feb 24;34(3):242. doi: 10.1007/s00520-026-10453-z (PMC12932401; doi:10.1007/s00520-026-10453-z)
Supplement: Supplementary file 1 — (DOCX 15.4 KB) [file 520_2026_10453_MOESM1_ESM.docx]

Appendix 2. Surgical outcome data.

|  | Oesophagectomy (n=7) | Laparotomy (n=10) | Laparoscopy (n=4) | All Patients  (n=21) |
| --- | --- | --- | --- | --- |
| Length of stay Mean (sd) | 17.4 (15.8) | 11.2 (4.2) | 7 (4.24) | 12.2 (10.1) |
| **Complications n (%)**  CD1  CD2  CD3  CD4  None | 1 (14.3)  3 (42.9)  0  2 (28.6)  1 (14.3) | 1 (10)  6 (60)  0  0  3 (30) | 0 (0)  0 (0)  0 (0)  0 (0)  4 (100) | 2 (9.5)  9 (42.9)  0 (0)  2 (9.5)  8 (38.1) |

SCC, squamous cell carcinoma; CD, Clavien-Dindo grade.
