# Supplementary material for: In-hospital moderate intensity interval training following surgical resection of foregut malignancy – a prospective single arm feasibility study
Source: Support Care Cancer. 2026 Feb 24;34(3):242. doi: 10.1007/s00520-026-10453-z (PMC12932401; doi:10.1007/s00520-026-10453-z)
Supplement: Supplementary file 2 — (DOCX 40.1 KB) [file 520_2026_10453_MOESM2_ESM.docx]

Appendix 3. Exercise intensity metrics by post-operative day and particular order of exercise

Table A3-1. Cohort’s average heart rate (mean, SD), matched percentage maximal heart rate (mean, SD), rating of perceived exertion /10 (mean, SD) by each post-operative day

|  | | Resistance Exercise 1 | Resistance Exercise 2 | Resistance Exercise 3 | Resistance Exercise 4 | Resistance Exercise 5 | Aerobic Exercise 1 | Aerobic Exercise 2 | Aerobic Exercise 3 |
| --- | --- | --- | --- | --- | --- | --- | --- | --- | --- |
| POD1 | Average HR | 86(11.5)  52.7(6.4)  3(2) | 81.9(9.8)  49.9(6.5)  4(1) | 94.9(1.6)  56.7(3.8)  3.5(1) | NA | NA | 85.3(15.9)  50.7(11.5)  6.5(3) | NA | NA |
|  | %Max. HR |  |  |  |  |  |  |  |  |
|  | RPE |  |  |  |  |  |  |  |  |
| POD2 | Average HR | 91.9(20.5)  56.7(12.5)  3.5(3) | 90.0(12.6)  55.4(5.9)  4(4) | 94.3(13.3)  57.1(5.1)  4(3) | NA | NA | 100.3(22.3)  60.2(10.3)  5(1) | 102.2(6.7)  63.6(7.9)  4.5(1) | NA |
|  | %Max. HR |  |  |  |  |  |  |  |  |
|  | RPE |  |  |  |  |  |  |  |  |
| POD3 | Average HR | 81.6(10.5)  51.5(6.5)  3.5(2) | 84.3(9.7)  52.7(6.4)  4.5(3) | 90.7(19.4)  57.3(12.5)  6(1) | 97.8(1.0)  61.1(3.4)  5(4) | 96.5(NA)  59.1(NA)  3(0) | 85.8(13.6)  52.8(7.1)  3(1) | 92.1(0.6)  56.2(2.4)  4(2) | NA |
|  | %Max. HR |  |  |  |  |  |  |  |  |
|  | RPE |  |  |  |  |  |  |  |  |
| POD4 | Average HR | 98.2(13.4)  53.5(9.5)  5(4) | 86.8(12.7)  52.5(7.6)  4(4) | 89.9(17.7)  53.6(10.8)  5(3) | 94.9(16.3)  56.9(9.1)  6(3) | NA | 107.6(18.6)  66.9(13.7)  4(3) | 101.6(NA)  60.4(NA)  8(NA) | NA |
|  | %Max. HR |  |  |  |  |  |  |  |  |
|  | RPE |  |  |  |  |  |  |  |  |
| POD5 | Average HR | 90.6(11.2)  57.7(7.7)  6(3) | 98.9(8.4)  62.8(6.3)  7(5) | 100.2(NA)  66.99(NA)  6(NA) | NA | NA | 93.9(3.6)  59.3(1.0)  3(4) | 106(NA)  64.7(NA)  8(NA) | NA |
|  | %Max. HR |  |  |  |  |  |  |  |  |
|  | RPE |  |  |  |  |  |  |  |  |
| POD6 | Average HR | 94.3(18.5)  59.7(10.4)  5(1) | 87.2(15.1)  55.6(8.0)  3.5(2) | 81.9(7.5)  53.2(4.8)  6(2) | 85.7(3.8)  56.1(1.4)  6(2) | 67(NA)  43.3(NA)  7(0) | 85.5(15.6)  54.4(8.3)  4.5(3) | 93.9(8.4)  59.7(3.0)  5.5(2.5) | 89.3(10.4)  55.2(5.4)  6.5(3) |
|  | %Max. HR |  |  |  |  |  |  |  |  |
|  | RPE |  |  |  |  |  |  |  |  |
| POD7 | Average HR | 91.1(10.0)  56.4(6.1)  5(1) | 86.9(8.9)  53.7(5.1)  6(1) | 91.2(8.1)  56.2(4.6)  5(3) | 90.3(10.9)  55.7(6.2)  5(3) | 98.5(13.8)  60.8(5.0)  5.5(3) | 94.8(17.9)  58.8(9.4)  3.5(2) | 93.2(27.0)  56.9(15.1)  8(2) | 103.9(1.6)  65.0(3.2)  7(4) |
|  | %Max. HR |  |  |  |  |  |  |  |  |
|  | RPE |  |  |  |  |  |  |  |  |
| POD8 | Average HR | 81.2(12.8)  51.1(7.0)  4.5(2.5) | 79.2(7.8)  49.8(3.6)  7(6) | 82.8(17.3)  51.5(9.1)  6.5(1) | 85.2(13.5)  53.1(6.6)  6.5(1) | 75(NA)  48.1(NA)  6(NA) | 96.8(NA)  61.7(NA)  4(NA) | 97(NA)  61.8(NA)  5(NA) | 102.5(NA)  65.3(NA)  7(NA) |
|  | %Max. HR |  |  |  |  |  |  |  |  |
|  | RPE |  |  |  |  |  |  |  |  |
| POD9 | Average HR | 90.3(3.8)  57.6(2.4)  4.5(1) | 91.3(5.7)  58.2(3.6)  3.5(1) | 90.7(NA)  57.8(NA)  3(NA) | NA | NA | NA | NA | NA |
|  | %Max. HR |  |  |  |  |  |  |  |  |
|  | RPE |  |  |  |  |  |  |  |  |
| POD10 | Average HR | 90.1(17.4)  57.4(9.2)  5(2) | 82.9(12.0)  53.0(7.2)  6(3) | 92.9(22.9)  59.0(11.6)  6(2.5) | 127.3(NA)  75.7(NA)  6(NA) | NA | 105.5(12.2)  67.0(6.6)  3(3) | 125(NA)  74.4(NA)  7(NA) | NA |
|  | %Max. HR |  |  |  |  |  |  |  |  |
|  | RPE |  |  |  |  |  |  |  |  |
| POD11 | Average HR | 85.7(18.2)  53.9(8.7)  6(3) | 87.0(22.3)  54.6(11.2)  4(3.5) | 91.4(33.4)  57.0(16.1)  6(2) | 121(NA)  72.0(NA)  8(NA) | NA | 86.1(23.7)  53.9(11.8)  4(4) | 19.3(3.8)  75.6(4.2)  6(4) | NA |
|  | %Max. HR |  |  |  |  |  |  |  |  |
|  | RPE |  |  |  |  |  |  |  |  |
| POD12 | Average HR | 97.0(NA)  60.7(NA)  3(NA) | 91.3(NA)  56.6(NA)  3(NA) | NA | NA | NA | 101.3(12.4)  63.2(4.9)  6.5(2.5) | 116(NA)  69.0(NA)  6(NA) | NA |
|  | %Max. HR |  |  |  |  |  |  |  |  |
|  | RPE |  |  |  |  |  |  |  |  |
| POD13 | Average HR | 77.3(NA)  52.1(NA)  4(NA) | 76.7(NA)  51.6(NA)  3(NA) | 78.0(NA)  52.5(NA)  4(NA) | NA | NA | 103.0(17.5)  65.9(6.8)  4(0) | 112.7(NA)  75.9(NA)  3(NA) | NA |
|  | %Max. HR |  |  |  |  |  |  |  |  |
|  | RPE |  |  |  |  |  |  |  |  |
| POD14 | Average HR | 75.7(NA)  51.0(NA)  4(NA) | 76.3(NA)  51.4(NA)  4(NA) | 77.3(NA)  52.1(NA)  4(NA) | NA | NA | 101.0(NA)  61.9(NA)  5(NA) | 119.4(NA)  73.2(NA)  5(NA) | NA |
|  | %Max. HR |  |  |  |  |  |  |  |  |
|  | RPE |  |  |  |  |  |  |  |  |

Percentage maximal heart rate was calculated for each individual, using formula stated in the article. Average heart rate and percentage maximal heart rate were recorded as means and standard deviations; rating of perceived exertion was recorded as median and interquartile range. POD, post-operative day; HR, heart rate; RPE, rating of perceived exertion; NA, not available.

Table A3-2 Highest percentage maximal heart rate by post-operative day in each individual patient

|  | POD1 | POD2 | POD3 | POD4 | POD5 | POD6 | POD7 | POD8 | POD9 | POD10 | POD11 | POD12 | POD13 | POD14 |
| --- | --- | --- | --- | --- | --- | --- | --- | --- | --- | --- | --- | --- | --- | --- |
| ID1 | X | R:58.5  A: NA | R:59.4  A:54.4 | X | X | X | R:61.1  A:NA | X | X | X | X | X | X | X |
| ID2 | X | X | X | X | R:49.8  A: NA | R:55.4  A:52.3 | X | X | X | X | X | X | X | X |
| ID3 | X | X | R:61.4  A:58.9 | R:69.9  A:64.0 | X | R:68.4  A:NA | R:64.4  A:67.0 | X | X | R:75.7  A:74.4 | R:72.0  A:72.6 | R:NA  A:69.0 | X | X |
| ID4 | R:59.3  A:NA | X | X | R:51.9  A:NA | R:55.0  A:NA | R:NA  A:58.5 | R:55.3  A:60.0 | X | X | X | X | X | X | X |
| ID5 | R:52.9  A:NA | R:52.1  A:NA | X | X | X | X | R:48.4  A:NA | R:54.8  A:65.3 | R:60.8  A:NA | X | X | X | X | X |
| ID6 | R:44.5  A:58.9 | X | X | R:50.9  A:NA | R:64.1  A:64.7 | R:NA  A:63.8 | R:56.7  A:68.5 | R:59.2  A:NA | X | X | X | X | X | X |
| ID7 | X | X | X | R:42.7  A:NA | X | X | X | X | X | X | X | X | X | X |
| ID8 | X | X | X | X | X | X | X | X | X | X | X | X | X | X |
| ID9 | X | X | R:54.0  A:NA | R:NA  A:62.9 | X | X | X | X | X | X | X | X | X | X |
| ID10 | X | X | X | X | X | X | X | R:46.5  A:NA | X | X | X | X | X | X |
| ID11 | R:67.2  A:NA | X | R:54.8  A:NA | R:47.8  A:NA | X | X | R:51.8  A:64.5 | X | X | X | X | X | X | X |
| ID12 | R:49.4  A:NA | R:57.7  A:64.8 | R:53.5  A:58.9 | R:57.3  A:62.5 | X | X | X | X | X | X | X | X | X | X |
| ID13 | R:55.6  A:NA | R:77.5  A:69.1 | R:52.6  A:NA | X | X | R:74.1  A:NA | R:60.9  A:NA | X | X | X | X | X | X | X |
| ID14 | X | R:46.4  A:NA | R:42.7  A:42.3 | X | X | R:49.3  A:59.5 | R:65.6  A:67.2 | R:49.1  A:NA | X | X | X | X | X | X |
| ID15 | X | X | R:48.6  A:57.9 | R:49.5  A:NA | R:55.9  A:NA | R:61.4  A:NA | R:NA  A:65.7 | X | X | R:56.6  A:65.7 | R:50.3  A:46.1 | R:60.7  A:65.7 | X | X |
| ID16 | X | X | X | X | X | R:50.7  A:61.4 | R:NA  A:61.4 | X | X | X | X | X | X | X |
| ID17 | X | X | X | X | R:NA  A:60.4 | R:51.2  A:56.6 | R:NA  A:46.5 | X | X | R:46.5  A:56.6 | R:47.1  A:78.6 | R:NA  A:57.2 | R:52.5  A:75.9 | R:52.1  A:NA |
| ID18 | R:55.7  A:NA | X | R:53.3  A:56.7 | R:NA  A:54.8 | X | X | X | X | R:59.3  A:NA | R:NA  A:70.7 | R:51.8  A:NA | X | X | X |
| ID19 | X | X | R:74.4  A:NA | R:72.4  A:94.1 | R:70.4  A:NA | X | X | X | X | R:64.4  A:74.0 | X | X | X | X |
| ID20 | R:51.5  A:NA | X | X | X | R:NA  A:58.8 | R:70.7  A:NA | R:60.5  A:NA | X | X | X | X | R:NA  A:61.4 | R:NA  A:70.7 | R:NA  A:73.2 |
| ID21 | R:54.0  A:42.6 | R:62.3  A:67.4 | X | R:56.7  A:63.3 | X | X | X | X | X | X | X | X | X | X |

Percentage maximal heart rate was calculated for each individual, using formula stated in the article; the highest number was recorded in this table as a percentage. POD, post-operative day, R, resistance exercise; A, aerobic exercise; NA, not available; X, not exercised.

Table A3-3 Rating of perceived exertion by post-operative day in each individual patient

|  | Baseline | POD1 | POD2 | POD3 | POD4 | POD5 | POD6 | POD7 | POD8 | POD9 | POD10 | POD11 | POD12 | POD13 | POD14 |
| --- | --- | --- | --- | --- | --- | --- | --- | --- | --- | --- | --- | --- | --- | --- | --- |
| ID1 | 4 | X | R:3  A:NA | R:3  A:2 | X | X | X | R:4  A:NA | X | X | X | X | X | X | X |
| ID2 | 7 | X | X | X | X | R:7  A: NA | R:6  A:9 | X | X | X | X | X | X | X | X |
| ID3 | 6 | X | X | R:6.5  A:6.5 | R:6.5  A:7 | X | R:3  A:NA | R:5  A:7 | X | X | R:6  A:6.5 | R:7  A:7.5 | R:NA  A:7.5 | X | X |
| ID4 | 3 | R:3  A:NA | X | X | R:3  A:NA | R:3  A:NA | R:NA  A:3 | R:5  A:5 | X | X | X | X | X | X | X |
| ID5 | 2 | R:3  A:NA | R:2  A:NA | X | X | X | X | R:5  A:NA | R:4  A:5 | R:4.5  A:NA | X | X | X | X | X |
| ID6 | 7 | R:6.5  A:8 | X | X | R:6  A:NA | R:6.5  A:7.5 | R:NA  A:7 | R:7.5  A:8 | R:7  A:NA | X | X | X | X | X | X |
| ID7 | 1 | X | X | X | R:3  A:NA | X | X | X | X | X | X | X | X | X | X |
| ID8 | 4 | X | X | X | X | X | X | X | X | X | X | X | X | X | X |
| ID9 | X | X | X | R:6  A:NA | R:NA  A:2 | X | X | X | X | X | X | X | X | X | X |
| ID10 | 3 | X | X | X | X | X | X | X | R:2  A:NA | X | X | X | X | X | X |
| ID11 | 5 | R:2  A:NA | X | R:3  A:NA | R:4  A:NA | X | X | R:5  A:5 | X | X | X | X | X | X | X |
| ID12 | X | R:3  A:NA | R:3  A:4 | R:2.5  A:3 | R:3  A:3 | X | X | X | X | X | X | X | X | X | X |
| ID13 | X | R:4.5  A:NA | R:5.5  A:5 | R:6  A:NA | X | X | R:7  A:NA | R:7  A:NA | X | X | X | X | X | X | X |
| ID14 | 6 | X | R:5  A:NA | R:7  A:4 | X | X | R:5  A:4.5 | R:5  A:5 | R:6  A:NA | X | X | X | X | X | X |
| ID15 | 3 | X | X | R:2.5  A:3 | R:7  A:NA | R:2.5  A:NA | R:5  A:NA | R:NA  A:3 | X | X | R:3  A:3 | R:5  A:3 | R:3  A:3 | X | X |
| ID16 | 3 | X | X | X | X | X | R:5  A:6 | R:NA  A:3 | X | X | X | X | X | X | X |
| ID17 | X | X | X | X | X | R:NA  A:3 | R:3  A:3 | R:NA  A:3 | X | X | R:7  A:NA | R:5  A:4 | R:NA  A:7 | R:4  A:3.5 | R:4  A:NA |
| ID18 | 3 | R:2  A:NA | X | R:2.5  A:3 | R:NA  A:3 | X | X | X | X | R:3  A:NA | R:NA  A:3 | R:3  A:NA | X | X | X |
| ID19 | 3 | X | X | R:5  A:NA | R:5.5  A:6 | R:6  A:NA | X | X | X | X | R:5  A:7 | X | X | X | X |
| ID20 | 3 | R:3  A:NA | X | X | X | R:NA  A:3 | R:5  A:NA | R:5.5  A:NA | X | X | X | X | R:NA  A:6 | R:NA  A:4 | R:NA  A:5 |
| ID21 | X | R:4  A:5 | R:5  A:5 | X | R:7  A:5 | X | X | X | X | X | X | X | X | X | X |

Rating of perceived exertion was recorded as median from each session of exercise for each individual. POD, post-operative day, R, resistance exercise; A, aerobic exercise; NA, not available; X, not exercised.
