# Supplementary material for: In-hospital moderate intensity interval training following surgical resection of foregut malignancy – a prospective single arm feasibility study
Source: Support Care Cancer. 2026 Feb 24;34(3):242. doi: 10.1007/s00520-026-10453-z (PMC12932401; doi:10.1007/s00520-026-10453-z)
Supplement: Supplementary file 3 — (PDF 216 KB) [file 520_2026_10453_MOESM3_ESM.pdf]

Exercise Program - Laparoscopy

Resistance/Functional Training

Position	Exercises			Intensity					Target Body Region
	**Variation 1**	**Variation 2**	**Variation 3**	POD1 (ICU/HDU)	POD2 (Ward)	POD3 (Ward)	POD4 (Ward)	POD5 (Ward)	
				Each session to include a 2 minute aerobic warm up; 1 min rest between sets					
Lying	Chest press with 1.5 kg dumbbells	Chest press with 3 kg dumbbells	Chest press with 5 kg dumbbells			2 sets of 6-15 reps RPE: 14-17	2 sets of 6-15 reps RPE: 14-17	3 sets 6-15 reps RPE: 14-17	upper body
	Knee extension - body weight	Knee extension with ankle weights		1 set of 6-15 reps RPE: 12-13	1 set of 6-15 reps RPE: 12-13	2 sets of 6-15 reps RPE: 14-17	2 sets of 6-15 reps RPE: 14-17	3 sets 6-15 reps RPE: 14-17	lower body
Sitting	Bicep curls with 1.5 kg dumbbells	Bicep curls with 3 kg dumbbells	Bicep curls with 5 kg dumbbells	1 set of 6-15 reps RPE: 12-13	1 set of 6-15 reps RPE: 12-13	2 sets of 6-15 reps RPE: 14-17	2 sets of 6-15 reps RPE: 14-17	3 sets 6-15 reps RPE: 14-17	upper body
	Shoulder press with 1.5 kg dumbbells	Shoulder press with 3 kg dumbbells	Shoulder press with 5 kg dumbbells			2 sets of 6-15 reps RPE: 14-17	2 sets of 6-15 reps RPE: 14-17	3 sets 6-15 reps RPE: 14-17	upper body
	Lateral raise with 1.5 kg dumbbells	Lateral raise with 3 kg dumbbells	Lateral raise with 5 kg dumbbells			2 sets of 6-15 reps RPE: 14-17	2 sets of 6-15 reps RPE: 14-17	3 sets 6-15 reps RPE: 14-17	upper body
	Sitting calf raises with 1.5 kg dumbbells	Sitting calf raises with 3 kg dumbbells	Sitting calf raises with 5 kg dumbbells	1 set of 6-15 reps RPE: 12-13	1 set of 6-15 reps RPE: 12-13	2 sets of 6-15 reps RPE: 14-17	2 sets of 6-15 reps RPE: 14-17	3 sets 6-15 reps RPE: 14-17	lower body
	Knee extension - body weight	Knee extension with ankle weights		1 set of 6-15 reps RPE: 12-13	1 set of 6-15 reps RPE: 12-13	2 sets of 6-15 reps RPE: 14-17	2 sets of 6-15 reps RPE: 14-17	3 sets 6-15 reps RPE: 14-17	lower body
Standing	Bicep curls with 1.5 kg dumbbells	Bicep curls with 3 kg dumbbells	Bicep curls with 5 kg dumbbells		1 set of 6-15 reps RPE: 12-13	2 sets of 6-15 reps RPE: 14-17	2 sets of 6-15 reps RPE: 14-17	3 sets 6-15 reps RPE: 14-17	upper body
	Lateral raise with 1.5 kg dumbbells	Lateral raise with 3 kg dumbbells	Lateral raise with 5 kg dumbbells			2 sets of 6-15 reps RPE: 14-17	2 sets of 6-15 reps RPE: 14-17	3 sets 6-15 reps RPE: 14-17	upper body
	Chair rises/Sit-to-stand from elevated bed	Chair rises/Sit-to-stand from chair			1 set of 6-15 reps RPE: 12-13	2 sets of 6-15 reps RPE: 14-17	2 sets of 6-15 reps RPE: 14-17	3 sets 6-15 reps RPE: 14-17	lower body
	Standing calf raises	Standing calf raises on step			1 set of 6-15 reps RPE: 12-13	2 sets of 6-15 reps RPE: 14-17	2 sets of 6-15 reps RPE: 14-17	3 sets 6-15 reps RPE: 14-17	lower body

"RPE - relative perceived exertion (out of 20)"

Aerobic Training

Position	Exercises	Intensity					Target Body Region
		POD1 (ICU/HDU)	POD2 (Ward)	POD3 (Ward)	POD4 (Ward)	POD5 (Ward)	
		Each session to include a 2 minute warm up and a 2 minute cool down at an RPE of 9-11.					
Lying	In-bed cycling with arm ergometer	5 x 15 sec (1 min rest) RPE: 12-13	5 x 30 sec (1 min rest) RPE: 12-13	5 x 1 min (1 min rest) RPE: 12-13	6 x 1 min (1 min slow) RPE: 14-17	7 x 1 min (1 min slow) RPE: 14-17	lower body
Sitting	Seated cycling with leg or arm ergometer	5 x 15-30 sec (1 min rest) RPE: 12-13	5 x 30 sec (1 min rest) RPE: 12-13	5 x 1 min (1 min rest) RPE: 12-13	6 x 1 min (1 min slow) RP: 14-17	7 x 1 min (1 min slow) RPE: 14-17	lower body
Standing	Walking		5 min RPE: 12-13	5-10 min RPE: 12-13	5-10 min RPE: 14-17	10-15 min RPE: 14-17	lower body
	Step ups			5 x 15 sec (1 min rest) RPE: 12-13	5 x 30 sec (1 min rest) RPE: 14-17	5 x 1 min (1 min rest) RPE: 14-17	lower body

When to terminate the exercise session:

- Onset or increase in pain (or pain medications)
- Muscle cramps or sudden onset of weakness or fatigue
- Disorientation, dizziness, blurred vision or fainting
- Sudden onset of nausea, vomiting
- Unusual or sudden shortness of breath
- Respiratory rate <8 or > 36/min
- SpO2 <90% (or <88% in COPD patient)
- Systolic arterial pressure > 180 mmHg
- Irregular heartbeat, palpitations, chest pain
- Changes in swelling/inflammation of the abdomen, groin or legs
- Numbness or loss of feelings in hands and/or feet
- Disconnection of tubes, intravenous lines, mechanical ventilation etc.
