# Supplementary material for: In-hospital moderate intensity interval training following surgical resection of foregut malignancy – a prospective single arm feasibility study
Source: Support Care Cancer. 2026 Feb 24;34(3):242. doi: 10.1007/s00520-026-10453-z (PMC12932401; doi:10.1007/s00520-026-10453-z)
Supplement: Supplementary file 4 — (PDF 168 KB) [file 520_2026_10453_MOESM4_ESM.pdf]

Exercise Program - Laparotomy

Resistance/Functional Training								
**Position**	**Exercises**			**Intensity**				**Target Body Region**
	**Variation 1**	**Variation 2**	**Variation 3**	POD1-2 (ICU/HDU)	POD3-4 (Ward)	POD5-6 (Ward)	POD7-8 (Ward)	
				Each session to include a 2 minute aerobic warm up; 1 min rest between sets				
Lying	Chest press with 1.5 kg dumbbells	Chest press with 3 kg dumbbells	Chest press with 5 kg dumbbells		2 sets of 6-15 reps RPE: 12-13	2 sets of 6-15 reps RPE: 14-17	3 sets 6-15 reps RPE: 14-17	upper body
	Knee extension - body weight	Knee extension with ankle weights		1 set of 6-15 reps RPE: 12-13	2 sets of 6-15 reps RPE: 12-13	2 sets of 6-15 reps RPE: 14-17	3 sets 6-15 reps RPE: 14-17	lower body
Sitting	Bicep curls with 1.5 kg dumbbells	Bicep curls with 3 kg dumbbells	Bicep curls with 5 kg dumbbells	1 set of 6-15 reps RPE: 12-13	2 sets of 6-15 reps RPE: 12-13	2 sets of 6-15 reps RPE: 14-17	3 sets 6-15 reps RPE: 14-17	upper body
	Shoulder press with 1.5 kg dumbbells	Shoulder press with 3 kg dumbbells	Shoulder press with 5 kg dumbbells		2 sets of 6-15 reps RPE: 12-13	2 sets of 6-15 reps RPE: 14-17	3 sets 6-15 reps RPE: 14-17	upper body
	Lateral raise with 1.5 kg dumbbells	Lateral raise with 3 kg dumbbells	Lateral raise with 5 kg dumbbells		2 sets of 6-15 reps RPE: 12-13	2 sets of 6-15 reps RPE: 14-17	3 sets 6-15 reps RPE: 14-17	upper body
	Sitting calf raises with 1.5 kg dumbbells	Sitting calf raises with 3 kg dumbbells	Sitting calf raises with 5 kg dumbbells	1 set of 6-15 reps RPE: 12-13	2 sets of 6-15 reps RPE: 12-13	2 sets of 6-15 reps RPE: 14-17	3 sets 6-15 reps RPE: 14-17	lower body
	Knee extension - body weight	Knee extension with ankle weights		1 set of 6-15 reps RPE: 12-13	2 sets of 6-15 reps RPE: 12-13	2 sets of 6-15 reps RPE: 14-17	3 sets 6-15 reps RPE: 14-17	lower body
Standing	Bicep curls with 1.5 kg dumbbells	Bicep curls with 3 kg dumbbells	Bicep curls with 5 kg dumbbells	1 set of 6-15 reps RPE: 12-13	2 sets of 6-15 reps RPE: 12-13	2 sets of 6-15 reps RPE: 14-17	3 sets 6-15 reps RPE: 14-17	upper body
	Lateral raise with 1.5 kg dumbbells	Lateral raise with 3 kg dumbbells	Lateral raise with 5 kg dumbbells		2 sets of 6-15 reps RPE: 12-13	2 sets of 6-15 reps RPE: 14-17	3 sets 6-15 reps RPE: 14-17	upper body
	Chair rises/Sit-to-stand from elevated bed	Chair rises/Sit-to-stand from chair			2 sets of 6-15 reps RPE: 12-13	2 sets of 6-15 reps RPE: 14-17	3 sets 6-15 reps RPE: 14-17	lower body
	Standing calf raises	Standing calf raises on step			2 sets of 6-15 reps RPE: 12-13	2 sets of 6-15 reps RPE: 14-17	3 sets 6-15 reps RPE: 14-17	lower body

"RPE - relative perceived exertion (out of 20)"

Aerobic Training

Position	Exercises	Intensity				Target Body Region
		POD1-2<br>(ICU/HDU)	POD3-4<br>(Ward)	POD5-6<br>(Ward)	POD7-8<br>(Ward)	
		Each session to include a 2 minute warm up and a 2 minute cool down at an RPE of 9-11.				
Lying	In-bed cycling with arm ergometer	5 x 15-30 sec<br>(1 min rest)<br>RPE: 12-13	5 x 1 min<br>(1 min rest)<br>RPE: 12-13	6 x 1 min<br>(1 min slow)<br>RPE: 14-17	7 x 1 min<br>(1 min slow)<br>RPE: 14-17	lower body
Sitting	Seated cycling with leg or arm ergometer	5 x 15-30 sec<br>(1 min rest)<br>RPE: 12-13	5 x 1 min<br>(1 min rest)<br>RPE: 12-13	6 x 1 min<br>(1 min slow)<br>RP: 14-17	7 x 1 min<br>(1 min slow)<br>RPE: 14-17	lower body
Standing	Walking		5-10 min<br>RPE: 12-13	5-10 min<br>RPE: 14-17	10-15 min<br>RPE: 14-17	lower body
	Step ups		5 x 15 sec<br>(1 min rest)<br>RPE: 12-13	5 x 30 sec<br>(1 min rest)<br>RPE: 14-17	5 x 1 min<br>(1 min rest)<br>RPE: 14-17	lower body

When to terminate the exercise session:

- Onset or increase in pain (or pain medications)
- Muscle cramps or sudden onset of weakness or fatigue
- Disorientation, dizziness, blurred vision or fainting
- Sudden onset of nausea, vomiting
- Unusual or sudden shortness of breath
- Respiratory rate <8 or > 36/min
- SpO2 <90% (or <88% in COPD patient)
- Systolic arterial pressure > 180 mmHg
- Irregular heartbeat, palpitations, chest pain
- Changes in swelling/inflammation of the abdomen, groin or legs
- Numbness or loss of feelings in hands and/or feet
- Disconnection of tubes, intravenous lines, mechanical ventilation etc
